# Genetic Mechanism Revealed of Age-Related Macular Degeneration Based on Fusion of Statistics and Machine Learning Method

**DOI:** 10.3389/fgene.2021.726599

**Published:** 2021-08-05

**Authors:** Yongyi Du, Ning Kong, Jibin Zhang

**Affiliations:** ^1^Department of Ophthalmology, Panyu Central Hospital, Guangzhou, China; ^2^Department of Stomatology, Panyu Central Hospital, Guangzhou, China

**Keywords:** AMD, GWAS, eQTL, SNPs, disease susceptibility

## Abstract

Age-related macular degeneration (AMD) is the most common cause of irreversible vision loss in the developed world which affects the quality of life for millions of elderly individuals worldwide. Genome-wide association studies (GWAS) have identified genetic variants at 34 loci contributing to AMD. To better understand the disease pathogenesis and identify causal genes for AMD, we applied random walk (RW) and support vector machine (SVM) to identify AMD-related genes based on gene interaction relationship and significance of genes. Our model achieved 0.927 of area under the curve (AUC), and 65 novel genes have been identified as AMD-related genes. To verify our results, a statistics method called summary data-based Mendelian randomization (SMR) has been implemented to integrate GWAS data and transcriptome data to verify AMD susceptibility-related genes. We found 45 genes are related to AMD by SMR. Among these genes, 37 genes overlap with those found by SVM-RW. Finally, we revealed the biological process of genetic mutations leading to changes in gene expression leading to AMD. Our results reveal the genetic pathogenic factors and related mechanisms of AMD.

## Introduction

Age-related macular degeneration (AMD) is the most common cause of irreversible blindness with limited therapeutic options in the elderly in many countries (Lim et al., 2012). AMD causes decreased photoreceptor function in the macular area of the retina (Fritsche et al., 2014). Researchers have found many factors which are related to the development and severity of AMD.

Genetic factors are significantly related to AMD. In 2005, Klein et al. found that CFH gene was related to AMD, which was the first discovered AMD-related gene (Haines et al., 2005). This gene is significantly expressed in retinal pigment epithelial cells. Y402H mutation of CFH impairs the complement pathway regulation function of CFH gene (Landowski et al., 2019). Subsequently, the ARMS2 gene cluster was also found to be related to AMD. Multiple studies have shown that there is a strong correlation between multiple genetic variants in this gene cluster and AMD (Johnson et al., 2001). Recently, it has been discovered that the apolipoprotein E (APOE) gene has a strong correlation with AMD (Fernández-Vega et al., 2020). The APOE gene plays a role in transporting lipids and cholesterol in the central nervous system, and multiple studies have shown that this gene is associated with neurodegenerative diseases such as Alzheimer’s disease and stroke (Feher et al., 2006; Zhao et al., 2019, 2020d). The gene is expressed on photoreceptor cells, retinal ganglion cells, retinal pigment epithelial cells, Bruch’s membrane, and the choroid. Most studies have proved APOE can prevent AMD (Pang et al., 2000). The genetic risk of advanced AMD is increased (Heiba et al., 1994). Researchers have found that the heritability estimate for twin studies is 0.45 for early AMD (Hammond et al., 2002) but 0.71 for late AMD (Seddon et al., 2005).

Computational methods have been widely used to discover functions of biological molecules (Zhao et al., 2020a,b, 2021a). AMD-related genome-wide association studies (GWAS) analyses have identified a strong association of 52 independent single-nucleotide polymorphisms (SNPs) at 34 genetic loci accounting for over 50% of the genetic heritability (Fritsche et al., 2016). Machine learning methods can help researchers find disease-related information on a large scale. However, these methods cannot explain the genetic mechanism of the results. GWAS studies are a valuable resource for understanding disease pathologies, but they may not precisely point out the causal genes responsible for the disease of interest. Besides, there have been studies that reported that causal genes are distinct from the nearest genes discovered by GWAS (Smemo et al., 2014; Claussnitzer et al., 2015). However, The gene expression is related to the genetic variant so the gene expression levels are different in different genotypes (Zhao et al., 2020c). Expression quantitative trait locus (eQTL) mapping offers a powerful approach to elucidate the genetic component underlying altered gene expression. Gene expression is vital for complex diseases (Zhao et al., 2021b) and is also differentially regulated across tissues, such as the brain, heart, and pancreas. Ratnapriya et al. (2019) have found potential causal genes in six AMD GWAS loci from human retinal samples. However, that analysis only considered retinal samples and was not comprehensive since it is difficult to obtain multiple living tissues and most eQTL studies so far have been performed with RNA isolated from immortalized lymphoblasts or lymphocytes. In this study, we fused random walk (RW) with support vector machine (SVM) to identify AMD-related genes. Since many GWAS and eQTL studies have been made public, to verify our results, AMD GWAS data and blood eQTL studies are integrated to further find expression of the genes related to AMD. In this method, we referred to the concept of Mendelian randomization (MR) analysis (Davey Smith and Ebrahim, 2003; Katan, 2004), where a genetic variant (such as a SNP) is considered as an instrumental variable (such as gene expression) to validate for the causative effect of an exposure on an outcome (such as a phenotype). Based on this assumption, we can obtain AMD-related genes based on MR. We collected eQTL data from the GTEx database and collected GWAS datasets including 12,711 advanced AMD cases and 14,590 controls of European descent from a study by Han et al. (2020); 707 Caucasian AMD patients and 2,014 controls from a study by Yan et al. (2018); and 14,034 cases, 91,214 controls, and 11 sources of data including the International AMD Genomics Consortium, IAMDGC, and United Kingdom Biobank (UKBB) from a study by Winkler et al. (2020). Based on these GWAS studies and eQTL dataset, we can not only identify genes related to AMD but also speculate on their biological processes.

## Materials and Methods

### Encoding Gene Interaction Network by Random Walk

The RW algorithm is a method that is simple to operate but not easy to fall into a local minimum. We constructed a gene interaction network by known AMD-related genes and a string database. Then, we implemented RW on the gene interaction network.

*f*(*x*) is a multivariate function with *n* variables; *x* = (*x*_1_,*x*_2_,…,*x*_*n*_) is an *n* dimension vector.

Step 1: Given the initial iteration point *x*, λ is the first walking step length, and ϵ is the control accuracy (ϵ is a very small positive number, used to control the end of the algorithm).

Step 2: Given the number of iterations control *N*, *k* is the current iteration number; set *k* = 1.

Step 3: When *k* < *N*, randomly generate an *n*-dimensional vector between (−1, 1). *u* = (*u*_1_,*u*_2_,…,*u*_*n*_), (−1 < *u*_*i*_ < 1, *i* = 1, 2,…, *n*), and standardize it to get *u*′.

$$u^{\prime }=\frac{u}{\sqrt{\sum u_{i}^{2}}}$$

Let *x*1 = x + λ*u*′ to complete the first step of walking.

Step 4: Calculate the value of the function, if *f*(*x*1) < *f*(*x*), which is a better point than the initial value, then reset *k* to 1, change *x*1 to *x*, and go back to step 2; otherwise, *k* = *k* + 1. Go back to step 3.

Step 5: If no better value can be found for *N* consecutive times, it is considered that the optimal solution is within the *N*-dimensional sphere with the current optimal solution as the center and the current step as the radius (if it is three-dimensional, it just happens to be in the space sphere). At this point, if λ < ϵ, the algorithm ends; otherwise, let λ = λ2, go back to step 1, and start a new round of walking.

Finally, we can get the gene feature after encoding the gene network.

### Classification by Support Vector Machine

**FIGURE 1:**
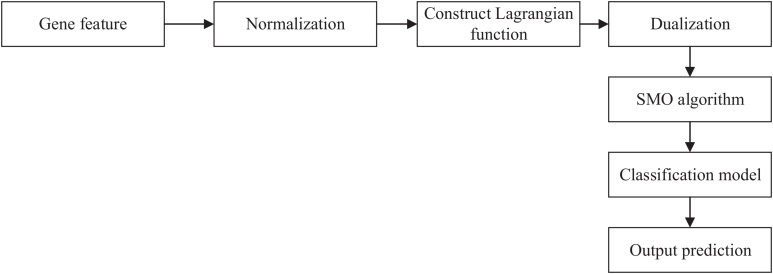
Workflow of SVM. SVM, support vector machine; SOM, sequential minimal optimization.

We obtained the gene feature in the last section. Then, we can input the gene feature and label into SVM to get the relationship between the gene and AMD. The workflow of SVM is shown in Figure 1.

First, we used *Z*-score normalization to process the gene feature. Then, we constructed a Lagrangian function to obtain the values and dualized the original problem. Sequential minimal optimization (SMO) algorithm was used to solve the dualization problem. Finally, we can obtain the classification model and output the prediction results.

## Results

### AMD-Related Genes Identification by SVM-RW

**FIGURE 2:**
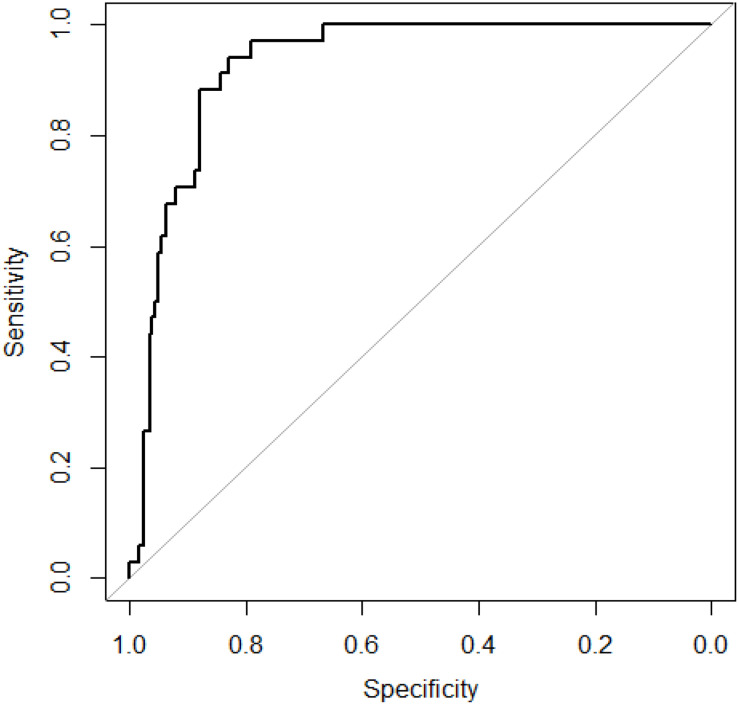
ROC curve of SVM-RW. ROC, receiver–operator characteristic; SVM-RW, support vector machine and random walk.

We obtained 34 known AMD-related genes from GWAS data. We constructed a gene network which has 239 nodes (genes). We did 10-cross validation by SVM-RW and tested the performance of SVM-RW. The area under the curve (AUC) of SVM-RW is shown in Figure 2.

SVM-RW achieved AUC of 0.927 in identifying AMD-related genes. We compared the results of SVM-RW with several other methods. The results are shown in Table 1.

**TABLE 1:** Comparison results.

Algorithm	AUC	AUPR
**SVM-RW**	**0.927**	**0.781**
Random forest-RW	0.852	0.645
Naive Bayes-RW	0.711	0.586
Backpropagation-artificial neural network-RW	0.823	0.692
Logistic regression-RW	0.691	0.531

*AUC, area under the curve; AUPR, area under the precision-recall curve; RW, random walk.*

*Bold values highlight the result of SVM-RW.*

After verifying the effectiveness of SVM-RW, we randomly selected 34 genes as negative samples and built a final SVM model. SVM-RW predicted 65 novel genes as AMD-related genes.

### Verify SVM-RW Results by Summary Data Level-Mendelian Randomization Analysis

If we use *g* to denote a genetic variant (such as a SNP), *x* as the expression level of a gene, and *y* as the trait, then the two-step least-squares (2SLS) estimate of the effect of *x* on *y* from an MR analysis can be denoted as:

(1) $${\hat{E}}_{x\,y}={\hat{E}}_{z\,y}/{\hat{E}}_{z\,x}$$

where ${\hat{E}}_{z\,y}$ and ${\hat{E}}_{z\,x}$ indicate the least-squares estimates of *y* and *x* on *z*, respectively, and *E*_*xy*_ indicates the effect size of *x* on *y* free of confounding from non-genetic factors. Then the sampling variance of the 2SLS estimate of *E*_*xy*_ can be denoted as:

(2) $$\text{var}\left({\hat{E}}_{x\,y}\right)=[\text{var}\left(y\right)\left(1-P_{x\,y}^{2}\right]/\left[n\text{var}\left(x\right)P_{z\,y}^{2}\right]$$

where *n* denotes the sample size, $P_{x\,y}^{2}$ indicates the proportion of variance in the explanation of *y* by *x*, and $P_{z\,y}^{2}$ is the proportion of variance in the explanation of *y* by *z*. Therefore, we use the statistic *T*_*MR*_ to test the significance of *E*_*xy*_; *T*_*MR*_ can be denoted as:

$$T_{M\,R}={\hat{E}}_{x\,y}^{2}/v\,a\,r\,\left({\hat{E}}_{x\,y}\right)$$

where T_*MR*_=$\chi_{1}^{2}$. Based on the suggestion that the power of detecting *E*_*xy*_ can be significantly increased using a two-sample MR analysis (Inoue and Solon, 2010; Pierce and Burgess, 2013), if GWAS and eQTL datasets share the same population, we can use unbiased estimates ${\hat{\epsilon }}_{z\,x}$ to replace *E*_*zx*_. We therefore have

(3) $${\hat{E}}_{x\,y}={\hat{E}}_{z\,y}/{\hat{\epsilon }}_{z\,x}$$

where ${\hat{E}}_{z\,y}$ is the estimate of a SNP effect from a GWAS for a trait, and ${\hat{\epsilon }}_{z\,x}$ is the estimate of a SNP effect on the expression level of a gene from an eQTL study. The sampling variance of ${\hat{E}}_{x\,y}$ can be approximately computed by the Delta method (Lynch and Walsh, 1998) as:

(4) $$\text{var}\,\left({\hat{E}}_{x\,y}\right)\approx \frac{E_{z\,y}^{2}}{\epsilon_{z\,x}^{2}}\,\left[\frac{\text{var}\,\left({\hat{\epsilon }}_{z\,x}\right)}{\epsilon_{z\,x}^{2}}+\frac{\text{var}\,\left({\hat{E}}_{z\,y}\right)}{{\text{E}}_{z\,y}^{2}}-\frac{2\,\text{cov}\,\left({\hat{\epsilon }}_{z\,x},{\hat{E}}_{z\,y}\right)}{\epsilon_{z\,x}\,E_{z\,y}}\right]$$

where $\text{cov}\,\left({\hat{\epsilon }}_{z\,x},{\hat{E}}_{z\,y}\right)$ is 0. Based on experience, we can replace them by their estimates in practice, indicated as an approximate χ^2^ test statistic of:

(5) $$T_{S\,M\,R}=\frac{{\hat{E}}_{x\,y}^{2}}{\text{var}\,\left({\hat{E}}_{z\,y}\right)}\approx \frac{z_{z\,y}^{2}\,z_{z\,x}^{2}}{z_{z\,y}^{2}+z_{z\,x}^{2}}$$

where *z*_*xy*_ is the *z* statistic of the GWAS and *z*_*zx*_ is the *z* statistic of the eQTL study.

**FIGURE 3:**
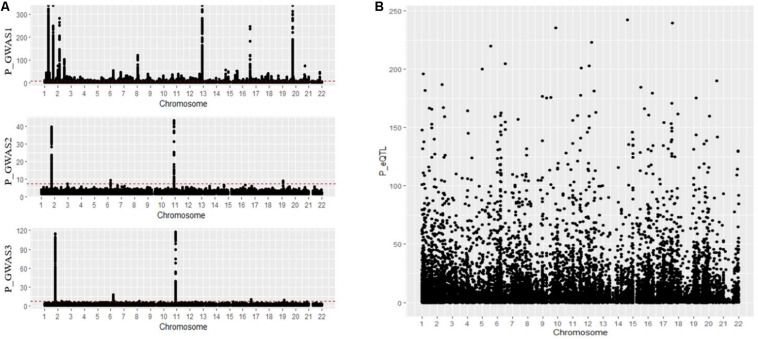
**(A,B)** Distribution of GWAS summary dataset and blood eQTL dataset. GWAS, genome-wide association studies; eQTL, expression quantitative trait locus.

In an MR analysis, *E*_*xy*_ is interpreted as the effect of a phenotype on the gene expression without considering non-genetic confounders. We first collected GWAS summary data and blood eQTL data from available online studies. We first collected a GWAS summary dataset composed of 12,711 advanced AMD cases and 14,590 controls of European descent from the study by Han et al. (2020); 707 Caucasian AMD patients and 2,014 controls from the study by Yan et al. (2018); and 14,034 cases, 91,214 controls, and 11 sourced from datasets including the International AMD Genomics Consortium, IAMDGC, and UKBB from the study by Winkler et al. (2020). The distribution of the above datasets is shown in Figures 3A,B.

**FIGURE 4:**
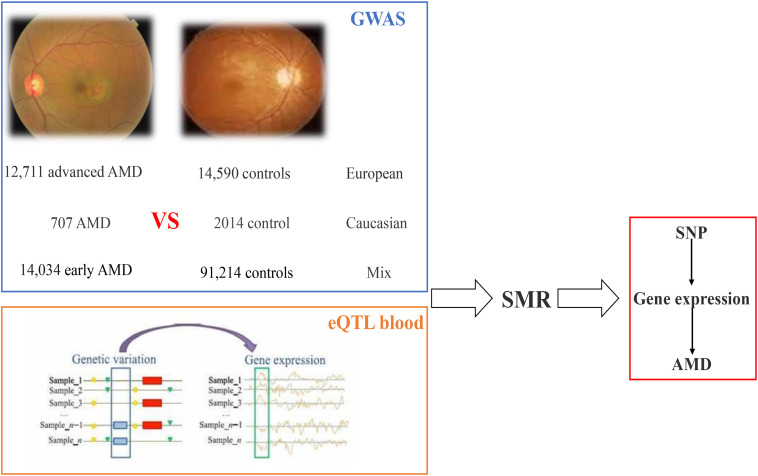
Workflow of SMR on AMD based on GWAS and eQTL datasets. AMD, age-related macular degeneration; GWAS, genome-wide association studies; eQTL, expression quantitative trait locus; SMR, summary data-based Mendelian randomization.

Then summary data-based Mendelian randomization (SMR) analysis is implemented on the blood eQTL data and GWAS data; in this paper, we identified 48 SNPs regulating 45 genes (including 41 coding genes and four non-coding genes) resulting in AMD susceptibility. The workflow is shown in Figure 4.

**FIGURE 5:**
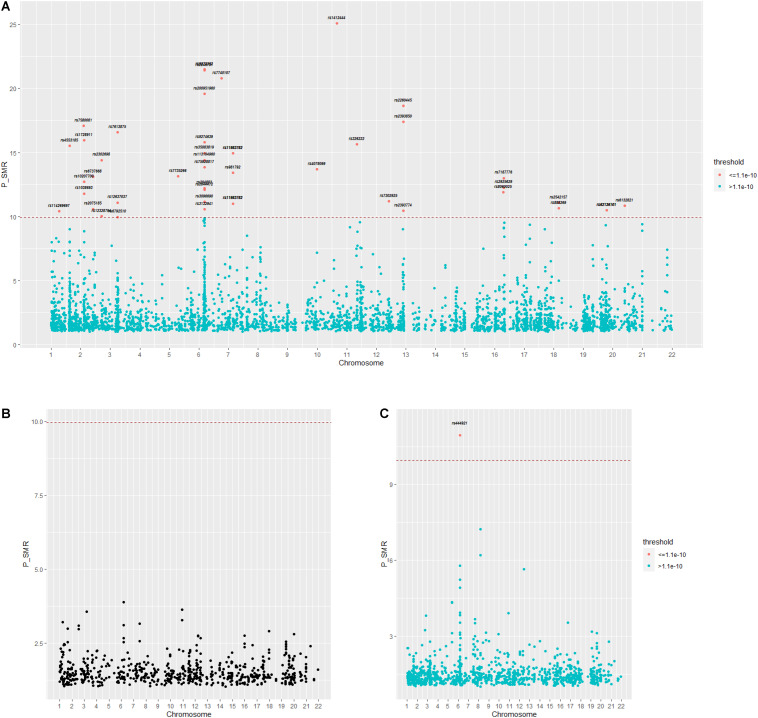
**(A–C)** Distribution of *p*-value calculated by SMR for three GWAS datasets. GWAS, genome-wide association studies; SMR, summary data-based Mendelian randomization.

For the first GWAS datasets consisting of 12,711 AMD cases and 14,590 controls from European cohorts, in total we found 3,872 SNPs coexist in both GWAS data and eQTL data; 43 of 3,872 SNPs are significant and regulate 44 genes in gene expression level. In the second GWAS dataset, we found 714 SNPs coexist in both GWAS dataset and eQTL dataset, with none significant. In the third GWAS dataset, we found 1,149 SNPs coexist both in GWAS dataset and eQTL dataset, with one significant regulating one gene in gene expression level. The distribution of the *p*-value of SNPs regulating genes tested by SMR is shown in Figures 5A–C. A Supplementary Table 1 indicates the *p*-values of significant SNPs regulating genes tested by SMR; the last line resulted from GWAS dataset 3, and the rest resulted from GWAS dataset 1.

### Case Study

Age-related macular degeneration has been described as a partly genetic disease (Heiba et al., 1994; Stone et al., 2004). Recently, a unifying hypothesis is that immune response gene polymorphisms modulate susceptibility to AMD. Human leukocyte antigen (HLA) polymorphisms, encoded within the major histocompatibility complex (MHC), are the most polymorphic within the human genome. In AMD, researchers detected intense HLA-DR immunoreactivity in not only soft but also hard drusen (Mullins et al., 2000). In the study of Goverdhan et al. (2005), considering the effect of smoking, age, and body mass index (BMI), HLA alleles B^∗^4001, DRB1^∗^1301, and Cw^∗^0701 were found to be related to AMD, which is consistent with our results displayed in Table 1.

In a study by Gu et al. (2013), they researched P2RX7 and P2RX4 genes in 744 AMD patients and 557 Caucasian controls and reached a conclusion that a rare functional haplotype of the P2RX4 leads to loss of innate phagocytosis and confers increased risk of AMD. P2RX7 and P2RX4 damage the normal scavenger function of macrophages and microglia through interaction, making individuals susceptible to AMD.

### Gene Interaction Network Based on AMD

**FIGURE 6:**
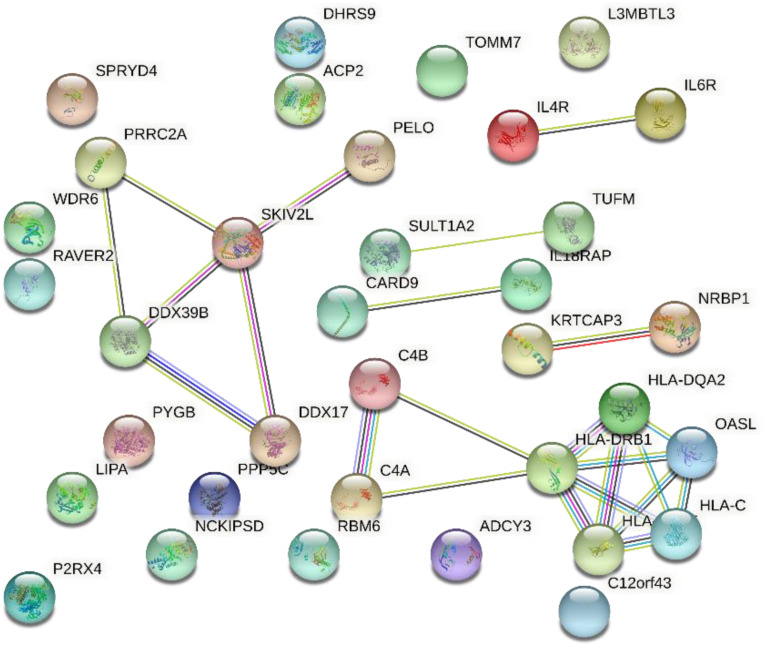
Gene interaction network obtained from 45 genes.

Figure 6 shows the gene interaction network produced from the results of SMR on AMD. Based on the interaction network, the HLA class intensively interacted and is significantly associated with AMD.

The cluster consisting of DDX39B (aka BAT1), PRRC2A (aka BAT2), and SKIV2L are genes found in the class III region of the MHC (MHC Class III). These genes encode RNA-binding proteins with clear roles in post-transcriptional gene regulation and RNA surveillance. They are likely to have important functions in immunity and are associated with autoimmune diseases (Schott and Garcia-Blanco, 2020). Early work by immunologists have shown that DDX39B promoted gene expression of anti-inflammatory pathways (Allcock et al., 2001). Therefore, understanding the genes interactions may help speculate on the proposed AMD mechanisms and immunotherapy.

## Conclusion

We applied the SMR method on AMD to test the gene–AMD associations based on GWAS summary data and blood eQTL data. From a total of 27,452 AMD cases and 107,818 controls, we obtained 44 SNPs regulating 45 genes significantly associated with AMD. Among the results, HLA class genes have been proved to be associated with immunologically mediated diseases because of the critical role of HLA in mediating the immune response, and genes from MHC Class III are also associated with autoimmune diseases. These genes may play important roles in causing AMD susceptibility and need to be further verified with experiments. Since AMD has been considered as a genetic disease, from this perspective, it is helpful in understanding the disease from gene-expression level to speculate about the AMD mechanisms and pathology and propose future treatment options for AMD.

## Data Availability Statement

The datasets presented in this study can be found in online repositories. The names of the repository/repositories and accession number(s) can be found in the article/Supplementary Material.

## Ethics Statement

Ethical review and approval was not required for the study on human participants in accordance with the local legislation and institutional requirements. Written informed consent for participation was not required for this study in accordance with the national legislation and the institutional requirements.

## Author Contributions

YD, NK, and JZ participated in its design, analyzed the data, and wrote the manuscript. All authors read and approved the published version of the manuscript.

## Conflict of Interest

The authors declare that the research was conducted in the absence of any commercial or financial relationships that could be construed as a potential conflict of interest.

## Publisher’s Note

All claims expressed in this article are solely those of the authors and do not necessarily represent those of their affiliated organizations, or those of the publisher, the editors and the reviewers. Any product that may be evaluated in this article, or claim that may be made by its manufacturer, is not guaranteed or endorsed by the publisher.
