# Supplementary material for: Genetic Mechanism Revealed of Age-Related Macular Degeneration Based on Fusion of Statistics and Machine Learning Method
Source: Front Genet. 2021 Aug 5;12:726599. doi: 10.3389/fgene.2021.726599 (PMC8375266; doi:10.3389/fgene.2021.726599)
Supplement: Supplementary file 1 [file Table_1.doc]

**Supplementary table**

*P*-value of Significant SNPs tested by SMR for AMD

| SNP | P-value | Gene |
| --- | --- | --- |
| rs1412444  rs9272362  rs2245731  rs7740107  rs200951960  rs2260445  rs2393850  rs7580081  rs7613875  rs1728911  rs9274639  rs1043402  rs326222  rs4553185  rs35083819  rs11983782  rs11983782  rs113104960  rs2302698  rs75928817  rs4078099  rs981792  rs7735266  rs6737668  rs7187776  rs10207700  rs2925629  rs204883  rs2844472  rs8060025  rs1038693  rs7302925  rs3096690  rs12637037  rs2542157  rs11983782  rs11983782  rs444921  rs6132821  rs888269  rs2075185  rs3132941  rs62136101  rs62136101  rs2393774  rs114269697  rs12328794  rs6792510 | 8.49e-26  3.27e-22  3.97e-22  1.68e-21  2.63e-20  2.35e-19  3.98e-18  8.5e-18  2.74e-17  1.15e-16  1.58e-16  1.59e-16  2.31e-16  3.04e-16  1.11e-15  1.16e-15  1.16e-15  3.84e-15  3.84e-15  1.44e-14  2e-14  3.94e-14  6.98e-14  7.69e-14  9.93e-14  1.98e-13  5.35e-13  5.94e-13  8.18e-13  1.29e-12  1.59e-12  6.47e-12  6.96e-12  7.98e-12  8.54e-12  9.64e-12  9.64e-12  1.110981e-11  1.45e-11  2.26e-11  2.6e-11  2.67e-11  3.22e-11  3.22e-11  3.54e-11  3.75e-11  9.29e-11  1.09e-10 | LIPA  HLA-DQA2  HLA-C  L3MBTL3  HLA-DRB1  OASL  P2RX4  ADCY3  RBM6  NRBP1  HLA-DQB1-AS1  DDX17  ACP2  IL6R  HLA-DRB5  AC005682.5  AC005682.5  DDX39B  DHRS9  HLA-DRB6  CARD9  TOMM7  PELO  IL18RAP  TUFM  KRTCAP3  SULT1A2  CYP21A1P  PRRC2A  IL4R  AC093690.1  SPRYD4  C4A  WDR6  LINC01882  AC005682.6  AC005682.6  SKIV2L  PYGB  RP11-973H7.1  AC007278.2  C4B  PPP5C  PPP5C  C12orf43  RAVER2  AC007556.3  NCKIPSD |
